# Etiology, Pathophysiology, and Treatment Strategies in the Prevention and Management of Metabolic Syndrome

**DOI:** 10.26502/aimr.0184

**Published:** 2024-10-28

**Authors:** Ritika Patial, Irene Batta, Manish Thakur, Ranbir Chander Sobti, Devendra K. Agrawal

**Affiliations:** 1 Centre for System Biology & Bioinformatics, Panjab University, Chandigarh, India; 2 Bothell High School, Bothell, Washington, USA; 3 School of Bioengineering & Biosciences, Lovely Professional University, Phagwara, Punjab, India; 4 Department of Biotechnology, Panjab University, Chandigarh, India; 5 Department of Translational Research, Western University of Health Sciences, Pomona, California, USA

**Keywords:** Cardiovascular diseases, Inflammation, Insulin resistance, Lipid metabolism, Metabolic Syndrome, Obesity, Type 2 diabetes

## Abstract

Metabolic Syndrome (MetS) is a complex cluster of metabolic irregularities that significantly increase the risk of developing chronic conditions, such as hypertension, type 2 diabetes, cardiovascular diseases, and other related disorders. This review aims to provide a comprehensive overview of the current understanding of MetS, its etiology and underlying pathogenesis, and the management strategies. MetS is characterized by central obesity, high blood pressure, insulin resistance, hyperglycemia, hypertriglyceridemia, and low high-density lipoprotein cholesterol levels. The prevalence of MetS is remarkably high, affecting approximately 25% of the global population, particularly in developed nations with inactive lifestyles and high-calorie diets. The development of MetS involves genetic and acquired factors, resulting in an inflammatory state that enhances the risk for cardiovascular disease. The biochemical alterations observed in MetS establish pathological connections between MetS, diabetes, and cardiovascular and neurodegenerative conditions. Despite its clinical importance, there is still debate regarding the precise components and pathophysiological associations among MetS elements. However, advancements in therapeutic measures, including drug therapies, surgical options, and experimental methods present promising avenues for managing and potentially reversing MetS. Further investigation of the MetS is critical because of its significant implications for public health and its connection to other clinical conditions and severe health outcomes, placing a substantial burden on healthcare system and society.

## Introduction

Metabolic Syndrome (MetS), also referred to as Syndrome X or obesity syndrome, is a complex cluster of metabolic irregularities that significantly enhances the probability of developing chronic conditions, such as type 2 diabetes (T2D), cardiovascular diseases (CVDs), and other related disorders [1-3]. This syndrome is characterized by central obesity, high blood pressure, insulin resistance, high blood sugar, increased triglycerides, and low high-density lipoprotein (HDL) cholesterol levels [1,4]. Insulin resistance plays a crucial role in MetS, connecting it to various metabolic processes and conditions including atherosclerotic cardiovascular disease and hepatic steatosis [1,5,6]. The development of MetS involves genetic and acquired factors, resulting in a final pathway of inflammation that worsens the CVD risk [1-4]. Indeed, several findings in the literature suggest that the risk of developing MetS starts from very early age, influenced by several factors, including child maltreatment [7], social and environmental determinants [8], and systemic inflammatory diseases [9]. The prevalence of MetS is remarkably high, affecting approximately 25% of the global population, especially in developed nations where inactive lifestyles and high-calorie diets are prevalent.

This syndrome is not a singular ailment, but a combination of interconnected risk factors that usually manifest together, posing a significant public health issue [10,11]. The biochemical alterations seen in MetS, such as disturbances in glucose and lipid metabolism, immune response, endothelial cell function, and intestinal microbiota, establish pathological connections between MetS, diabetes, and cardiovascular and neurodegenerative conditions [1,12-14]. Indeed, the cardiac autonomic regulation worsens with the increase in the number of factors associated with MetS [15]. Despite its clinical importance, there is still discussion concerning the precise components and pathophysiological associations among the elements of MetS, limiting its effectiveness as a clinical tool [1,2,4]. Nevertheless, progress in the identification of biomarkers [16-18], therapeutic measures, including prescribed drugs and over-the-counter medicine [19-23], surgical choices like bariatric surgery, and experimental methods such as gene therapy, present hopeful strategies for handling and potentially reversing MetS [24-27]. The intricate nature of the syndrome is further emphasized by its close relationship with human behavior and evolution, highlighting the significance of diet, inflammation, and hormonal regulation in its onset and persistence [1,4,8]. Early identification and adjustments in lifestyle and control on the body weight are critical for reducing the health hazards associated with MetS, underscoring the necessity for a thorough understanding of its epidemiology, diagnostic criteria, and management approaches [11,28].

## Importance of Studying Metabolic Syndrome

Detailed investigation of MetS is extremely important because of its significant implications for public health and its connection to severe health outcomes. It is characterized by a combination of metabolic disturbances, including central obesity, high blood pressure, hyperglycemia, abnormal lipid profiles, and insulin resistance, which collectively increase the risk of cardiovascular disease (CVD), type 2 diabetes, and other related disorders [1-4]. The prevalence of MetS is remarkably high and continues to increase globally, reflecting the obesity epidemic and sedentary lifestyles, with rates ranging from 44.9% in Japan to 50.9% in Spain [29]. This syndrome not only impacts individual health but also places a significant burden on healthcare systems and society, leading to higher disability and mortality rates. The association between MetS and cardiovascular disease is particularly concerning, as MetS significantly increases the chances of developing CVD and type II diabetes, highlighting the need for a thorough understanding of its risk factors and prevalence to guide effective prevention and management strategies [1,4-6]. Moreover, MetS is associated with various health issues, such as diabetes [1,30], acute coronary syndrome [31], thyroid sensitivity [32], rheumatoid arthritis [3], psoriasis vulgaris [33], musculoskeletal disorders [34], osteoarthritis [35], cognitive functions [36], Parkinson’s disease [37], and non-alcoholic fatty liver disease [5,6], owing to dietary pattern and gut microbiota [10], and many biochemical and pathophysiological changes including hyperglycemia [1,16], hyperlipidemia [38-41], increased oxidative stress [42], systemic inflammation [5, 16-18], and autonomic dysregulation [15] . These co-morbidities, risk factors, and pathophysiological changes may trigger MetS.

**Figure 1:**
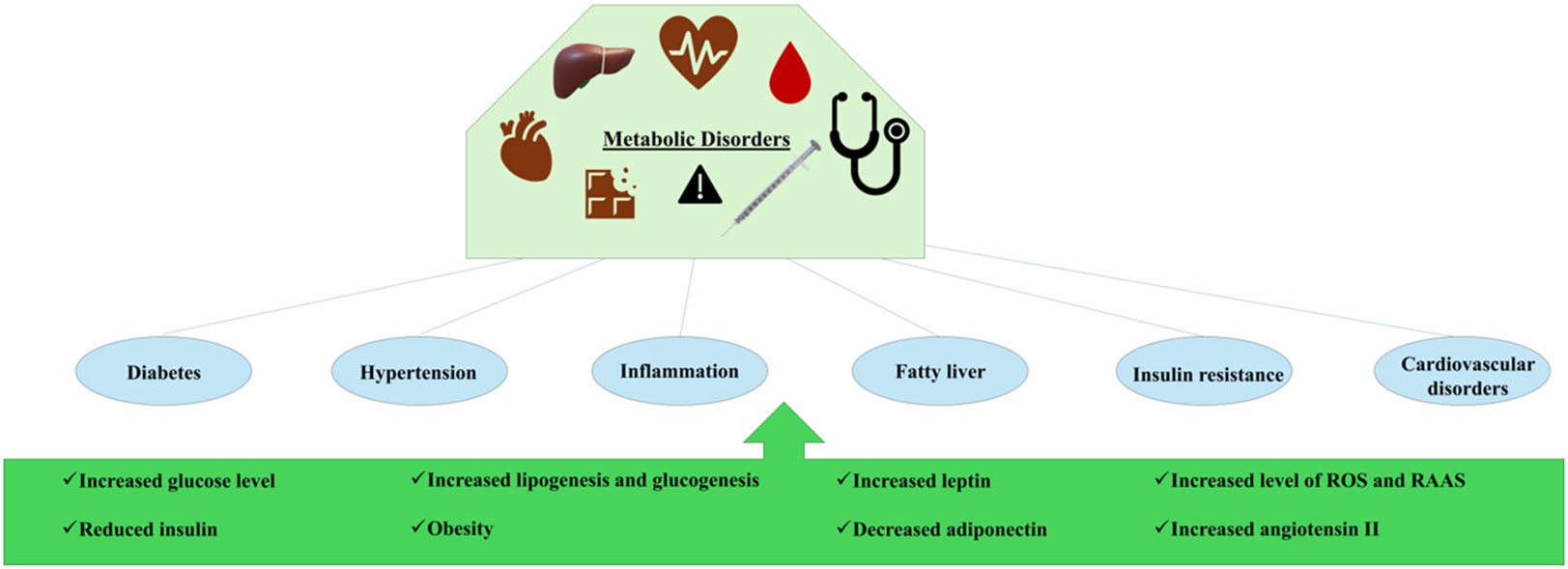
Pathophysiology of metabolic syndrome. This figure illustrates the complex interplay between genetic, lifestyle, and dietary factors in the development of metabolic disorders, highlighting the cascade of physiological changes that lead to significant health conditions. RAAS, renin-angiotensin-aldosterone system; ROS, reactive oxygen species.

The pathophysiology of MetS involves an intricate interplay between genetic, lifestyle, dietary, and environmental factors (Figure 1). A clear understanding these mechanisms is vital for creating successful prevention and treatment approaches. Additionally, MetS is linked to persistent low-grade inflammation and inflammatory cytokine activity, which leads to organ damage and elevated biomarkers in the bloodstream, underscoring the importance of better understanding inflammatory triggers and organ interference to identify therapeutic targets [16-27]. Public health efforts and personalized preventive measures are crucial in addressing the escalating prevalence of MetS, stressing the significance of promoting healthy lifestyles, including regular physical activity, balanced diets, and avoiding harmful habits such as smoking and excessive alcohol consumption [28]. Also, abdominal obesity could be interpreted as metabolically healthy or metabolically unhealthy obesity. Recent findings suggest that serum calcium levels could be used as a biomarker in the assessment and diagnosis of unhealthy versus healthy obesity [43].

Innovative therapeutic interventions, such as pharmacological treatments, surgical options such as bariatric surgery, and emerging experimental techniques such as gene therapy, present promising avenues for managing MetS and lessening its global impact [24,25]. The urgency of dealing with MetS is emphasized by its role as a critical precursor to major health conditions, requiring proactive measures to minimize its impact on public health and to protect the well-being of populations worldwide. Thus, comprehensive interdisciplinary approaches are necessary to effectively address this global health challenge, making the study of MetS a vital pursuit in contemporary healthcare research and practice.

## Current Understanding of Metabolic Syndrome

A current understanding of metabolic syndrome is important because it allows healthcare professionals to stay updated on the latest research, diagnostic criteria, and treatment options. This knowledge is crucial for the accurate diagnosis, effective management, and prevention of complications associated with metabolic syndromes, such as heart disease, diabetes, and stroke. By staying informed with latest knowledge, healthcare providers can provide better care and support to individuals at risk or already diagnosed with metabolic syndrome. Furthermore, ongoing education and training in the field of metabolic syndromes enables healthcare professionals to implement evidence based practices and tailor interventions to meet the specific needs of each patient, ultimately improving outcomes and quality of life.

## Etiology of Metabolic Syndrome

The origin of metabolic disorders is complex and involves genetic, environmental, and lifestyle factors that disrupt the normal metabolic processes. Inherited metabolic disorders, also called inborn errors of metabolism, result from single-gene defects causing enzyme deficiencies, which disturb metabolic balance and lead to conditions such as gout, Lesch-Nyhan syndrome, and citrullinemia [44-46]. These disorders typically appear as abnormal chemical reactions in the body, affecting the metabolism of macronutrients, such as proteins, fats, and carbohydrates. Genetic mutations can also affect crucial regulatory pathways, such as the JAK-STAT signaling cascade, implicated in insulin resistance and hepatic steatosis, contributing to obesity and type 2 diabetes [48]. Environmental influences, such as poor dietary choices and sedentary lifestyles, worsen these conditions by upsetting metabolic equilibrium and generating harmful substances that may initiate disrupted metabolic signals, resulting in the pathogenesis of MetS [49,50]. Obesity, a major risk factor for metabolic disorders, interferes with intracellular insulin signaling by producing inflammatory substances and adipokines, resulting in insulin resistance, high blood sugar, high blood pressure, and abnormal lipid levels [1-3]. Additionally, oxidative stress, alterations in miRNA expression, and epigenetic changes are common pathological mechanisms underlying metabolic disorders, such as diabetes mellitus, dyslipidemia, and osteoporosis [37-41]. Research on rare monogenic disorders has shed light on the molecular basis of common metabolic syndromes, underscoring the impact of single-gene defects on severe obesity, early onset diabetes, and insulin resistance [44, 45]. Furthermore, chronic ailments such as osteomyelitis can disrupt metabolic balance by affecting the equilibrium between bone formation and breakdown, requiring etiopathogenetic therapy to address these metabolic disruptions and enhance treatment outcomes [51]. The accumulation of metabolites, such as hypoxanthine and citrulline, in amyloid structures highlights the intricate biochemical nature of metabolic disorders, indicating a shared etiological connection in IEMs and presents potential targets for therapeutic interventions [52].

## Criteria Established for Metabolic Syndrome by Different Organizations

The assessment and diagnosis of metabolic diseases such as metabolic syndrome have been the subject of ongoing discussions and debates within the medical community. Various organizations have proposed criteria to help clinicians effectively identify and manage these complex conditions (Table 1).

## Insulin Resistance

Metabolic syndrome comprises a group of conditions that increase the likelihood of heart disease, stroke, and diabetes. It is characterized by insulin resistance, obesity, dyslipidemia, and hypertension. Insulin resistance, a fundamental element of metabolic syndrome, occurs when the cells of the body become less receptive to insulin, resulting in increased blood glucose levels [1-4]. The pathophysiology of metabolic syndrome and insulin resistance involves an intricate interaction between genetic, environmental, and metabolic factors (Figure 1). One of the main mechanisms consists of the disruption of adipose tissue function. Adipose tissue, particularly visceral fat, is pivotal in insulin resistance development [53,54]. It releases various adipokines and cytokines that can trigger an inflammatory state, contributing to insulin resistance and metabolic syndrome [1-3]. Chronic inflammation significantly contributes to the development of insulin resistance. Inflammatory cytokines, such as TNF-α and IL-6, which are often elevated in obese individuals, hinder insulin signaling pathways, causing impaired glucose absorption by cells [55]. Oxidative stress worsens this inflammatory condition, which further harms cellular components and hampers normal metabolic processes [37,41]. Another crucial aspect is the involvement of the liver in metabolic syndromes. Non-alcoholic fatty liver disease (NAFLD) is frequently linked to insulin resistance and is viewed as both a cause and consequence of metabolic syndrome. The inability of the liver to metabolize lipids effectively results in increased free fatty acids in the blood, which can worsen insulin resistance in peripheral tissues [5,6]. Genetic predisposition also influences insulin resistance and the development of metabolic syndrome. Specific genetic variations can affect insulin signaling pathways, lipid metabolism, and adipokine production, predisposing individuals to these conditions [44, 56]. Nonetheless, lifestyle elements, such as diet and physical activity, play a vital role in moderating these genetic risks. A sedentary lifestyle and a high-calorie diet can lead to obesity, which is a significant risk factor for insulin resistance [27]. The interaction between these elements establishes a harmful cycle in which insulin resistance leads to hyperinsulinemia, further boosting lipid accumulation and inflammation, thereby exacerbating insulin resistance. This cycle emphasizes the importance of early intervention through lifestyle adjustments, and when needed, pharmaceutical interventions to control and prevent the advancement of metabolic syndrome and its associated complications.

## Dyslipidemia

Dyslipidemia, a critical element of metabolic syndrome, is characterized by irregular lipid levels in the bloodstream, including heightened triglycerides, reduced high-density lipoprotein (HDL) cholesterol, and occasionally increased low-density lipoprotein (LDL) cholesterol [57]. Dyslipidemia in metabolic syndrome is influenced by intricate connections among genetic, environmental, and metabolic factors. The primary mechanism underlying dyslipidemia in metabolic syndrome is insulin resistance, a hallmark of the syndrome. Insulin resistance affects lipid metabolism by hindering the typical function of insulin in adipose tissue, leading to increased lipolysis and the discharge of free fatty acids into the bloodstream. This process contributes to the excessive production of very low-density lipoprotein (VLDL) in the liver, culminating in hypertriglyceridemia [58, 59]. Moreover, insulin resistance correlates with reduced activity of lipoprotein lipase, a critical enzyme for breaking down triglycerides in lipoproteins, thereby worsening hypertriglyceridemia [59]. Another notable aspect is the significance of adipokines, which are secreted by adipose tissue. In metabolic syndrome, there is frequently an imbalance in adipokines, including heightened levels of pro-inflammatory cytokines such as tumor necrosis factor-alpha (TNF-α) and interleukin-6 (IL-6), along with decreased levels of adiponectin, an anti-inflammatory cytokine [1-4]. This imbalance contributes to systemic inflammation, exacerbates insulin resistance, and perpetuates the dyslipidemia cycle. Genetic predisposition also plays a vital role in the onset of dyslipidemia in metabolic syndrome. Variations in genes related to lipid metabolism, such as those responsible for apolipoproteins and enzymes like hepatic lipase, can affect lipid levels and the likelihood of developing dyslipidemia [44]. Additionally, environmental factors, including diet and physical activity, substantially influence lipid profiles. Diets rich in saturated fats and sugars can worsen dyslipidemia, whereas routine physical activity can enhance lipid levels by boosting insulin sensitivity and facilitating triglyceride removal from the bloodstream [27]. Furthermore, oxidative stress has been implicated in the pathophysiology of dyslipidemia in metabolic syndrome. Elevated oxidative stress can trigger the oxidation of LDL particles, rendering them more atherogenic and contributing to the progression of atherosclerosis, a frequent complication of dyslipidemia [1,37]. Antioxidant defenses are frequently compromised in individuals with metabolic syndrome, which further intensifies oxidative damage and lipid irregularities [41].

## Hypertension

**Figure 2:**
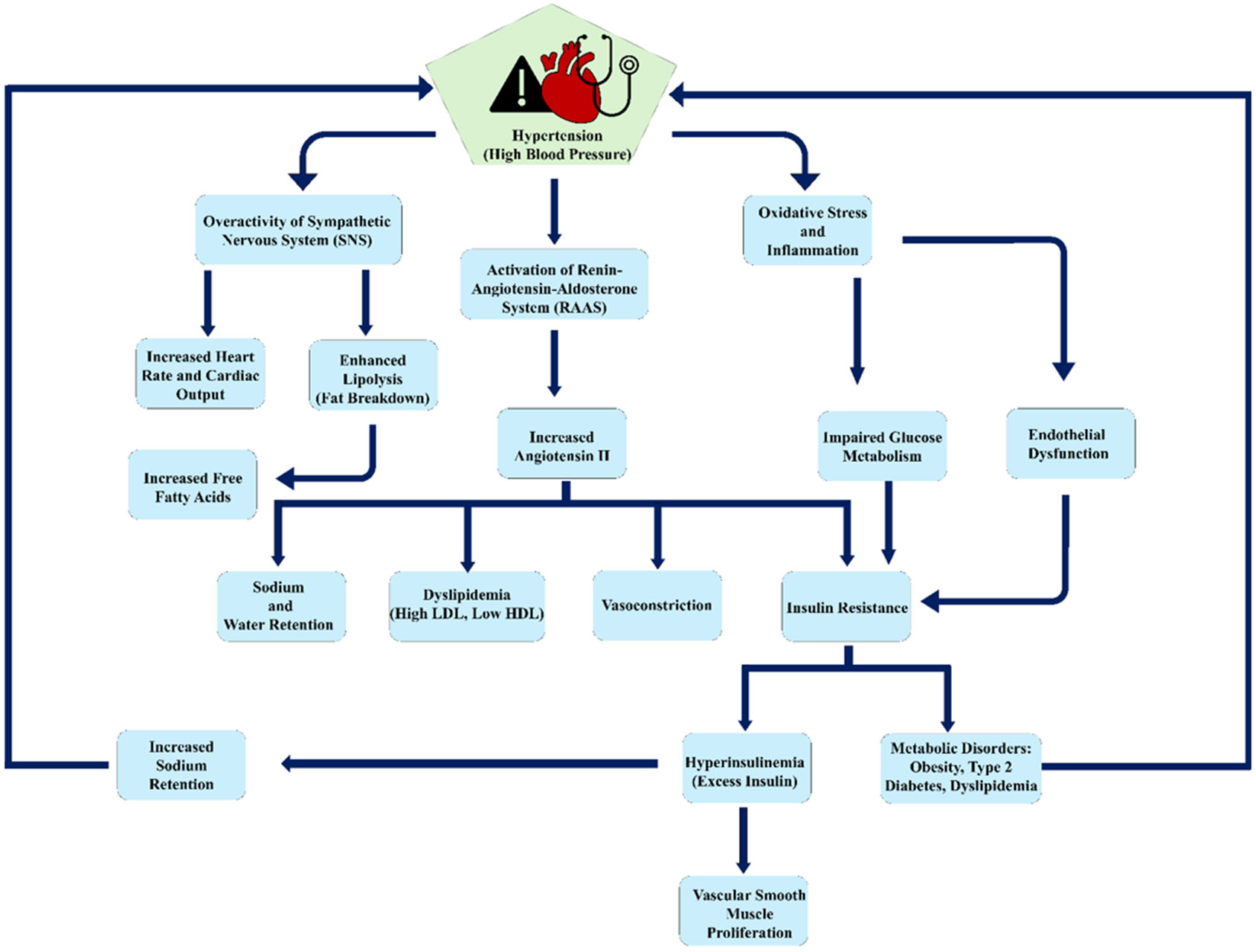
Interconnected pathways of hypertension leading to metabolic disorders. The pathways highlight the role of the Renin-Angiotensin-Aldosterone System (RAAS), insulin resistance, obesity, and inflammation. These pathways collectively contribute to conditions like diabetes and cardiovascular complications.

Metabolic syndrome is defined by a group of conditions such as insulin resistance, obesity, dyslipidemia, and hypertension, which collectively increase the risk of cardiovascular diseases and type 2 diabetes. One of the key mechanisms connecting metabolic syndrome to hypertension is insulin resistance, which is a key feature of metabolic syndrome that contributes to hypertension through multiple pathways. This leads to hyperinsulinemia, which can enhance sympathetic nervous system activity and encourage vasoconstriction and sodium retention, thereby increasing blood pressure (Figure 2) [60, 61]. Furthermore, insulin resistance hampers endothelial function, reducing the availability of nitric oxide, a potent vasodilator, which further contributes to increased vascular resistance and hypertension [62]. Obesity, especially visceral adiposity, is another crucial element in the pathophysiology of hypertension in metabolic syndrome. Excessive adipose tissue acts as an endocrine organ, releasing various adipokines and inflammatory cytokines that can impact blood pressure regulation. For example, elevated levels of leptin, an adipokine, can boost sympathetic nervous system activity, while reduced levels of adiponectin, which possesses anti-inflammatory and vasodilatory properties, are linked to hypertension [1,63]. Additionally, obesity is associated with increased renin-angiotensin-aldosterone system (RAAS) activity, which encourages sodium retention and vasoconstriction, contributing to elevated blood pressure [64]. Dyslipidemia, another component of the metabolic syndrome, also plays a role in the development of hypertension. High triglyceride and low high-density lipoprotein (HDL) cholesterol levels can result in endothelial dysfunction and increased arterial stiffness, both of which are associated with higher blood pressure [1]. Oxidative stress from dyslipidemia further worsens endothelial dysfunction and promotes hypertension [37,41]. Inflammation is a common underlying factor of metabolic syndrome that contributes to hypertension. Persistent low-grade inflammation, marked by elevated levels of inflammatory markers such as C-reactive protein (CRP) and interleukin-6 (IL-6), is common in individuals with metabolic syndrome [65,66]. This inflammatory condition can lead to endothelial dysfunction and increased arterial stiffness, both of which are crucial for the pathogenesis of hypertension.

## Obesity

The pathophysiology of obesity-related metabolic syndrome involves a complicated interaction between genetic, environmental, and physiological elements that leads to the onset of this condition. Metabolic syndrome is identified by a combination of factors that collectively increase the risk of heart disease, stroke, and type 2 diabetes. At the core of the pathophysiology of metabolic syndrome, obesity is insulin resistance, a state in which the cells of the body exhibit reduced sensitivity to insulin, resulting in increased blood glucose levels. Insulin resistance is frequently worsened by obesity, especially visceral fat accumulation, which pertains to fat storage in the abdominal area. This fat is actively metabolized and releases free fatty acids, inflammatory cytokines, and various bioactive compounds that promote systemic inflammation and further insulin resistance [1,16]. Adipose tissue dysfunction is a crucial factor in the pathophysiology of obesity-related metabolic syndrome. In obese individuals, adipose tissue experiences both hypertrophy and hyperplasia, resulting in a disrupted secretion pattern of adipokines including leptin and adiponectin [1,63]. Leptin resistance, which is prevalent in obesity, hinders the regulation of hunger and energy balance, whereas lower levels of adiponectin correlate with heightened insulin resistance and inflammation. Chronic low-grade inflammation is also essential for the emergence of metabolic syndrome [63]. Obesity is linked to greater infiltration of immune cells, such as macrophages, into adipose tissue, which fosters a pro-inflammatory environment. Inflammation is associated with the onset of insulin resistance and various metabolic irregularities typical of metabolic syndrome [4]. Genetic predisposition is another element that affects the pathophysiology of metabolic syndrome. Specific genetic variations can influence fat distribution, insulin sensitivity, and lipid metabolism, making individuals more susceptible to metabolic syndrome [44]. Nonetheless, environmental factors, including diet and exercise, are equally important in moderating these genetic vulnerabilities. Additionally, gut microbiota has been recognized as a factor in the pathophysiology of metabolic syndrome obesity [10]. Dysbiosis, or an imbalance within the gut microbial ecosystem, can affect energy extraction from food, fat accumulation, and inflammation, thereby contributing to obesity and metabolic syndromes.

## Complications Associated with Metabolic Syndrome

Metabolic syndrome, a cluster of conditions characterized by insulin resistance, abdominal obesity, dyslipidemia, and hypertension, has become increasingly prevalent in modern societies. These syndromes pose significant public health challenges, as they are associated with a three-fold increase in the risk of type 2 diabetes and a two-fold increase in the risk of cardiovascular disease.

### A. Cardiovascular Diseases:

Metabolic syndrome considerably increases the risk of cardiovascular disease (CVD). This syndrome manifests as a blend of hypertension, dyslipidemia, insulin resistance, and central obesity, all of which are recognized risk factors for CVD. Research has revealed that those with metabolic syndrome exhibit a greater prevalence of atherosclerosis, potentially leading to coronary artery disease and other cardiovascular issues [1-3]. Inflammatory reactions and oxidative stress linked to metabolic syndrome further increase cardiovascular risk by fostering endothelial dysfunction and plaque buildup in the arteries [37,67,68].

### B. Type 2 Diabetes:

Type 2 diabetes is a significant complication associated with metabolic syndrome. Insulin resistance, a fundamental aspect of metabolic syndrome, is a precursor to the onset of type 2 diabetes. Research indicates that the presence of metabolic syndrome markedly increases the chance of developing type 2 diabetes due to disrupted glucose metabolism and pancreatic beta-cell failure [1-4]. The persistent low-grade inflammation found in metabolic syndrome also plays a role in the development of type 2 diabetes by hindering insulin signaling pathways.

### C. Non-Alcoholic Fatty Liver Disease (NAFLD):

NAFLD is closely associated with metabolic syndrome and insulin resistance is pivotal to its pathophysiology. The buildup of fat within liver cells, a defining feature of NAFLD, is frequently observed in individuals with metabolic syndrome due to unregulated lipid metabolism [5, 6]. NAFLD has the potential to evolve into more severe liver diseases, including nonalcoholic steatohepatitis (NASH), cirrhosis, and hepatocellular carcinoma. The occurrence of NAFLD is significantly greater among patients with metabolic syndrome, highlighting the necessity for timely detection and management of metabolic risk factors to avert liver-related complications [5].

### D. Stroke:

Stroke is another serious complication associated with metabolic syndrome. The likelihood of both ischemic and hemorrhagic strokes escalates in individuals with metabolic syndrome owing to the cumulative effects of hypertension, dyslipidemia, and hyperglycemia, all of which contribute to cerebrovascular harm. The pro-thrombotic condition instigated by metabolic syndrome further increases the risk of stroke by facilitating clot formation in the cerebral arteries [69-71]. Moreover, the inflammatory environment associated with metabolic syndrome can result in vascular remodeling and heightened arterial stiffness, which further increases the risk of stroke.

## Management and Treatment Strategies

Management and treatment strategies for a range of health conditions include lifestyle changes, pharmacological therapies, and surgical procedures. Each method presents unique advantages and obstacles, and its effectiveness may differ depending on the conditions being addressed.

### A. Lifestyle Interventions:

Lifestyle modifications are essential for the management of chronic diseases, especially those associated with metabolic health. These changes usually involve alterations in diet, increased physical activity, and behavioral shifts. For example, research underscores the significance of dietary modifications in managing metabolic syndrome, highlighting how a balanced diet abundant in fruits, vegetables, and whole grains can enhance metabolic health indicators [27]. Furthermore, engaging in regular physical exercise is vital for tackling obesity and related issues as it aids in weight loss and promotes better cardiovascular health [29]. Behavioral strategies, including cognitive-behavioral therapy, can also facilitate lifestyle changes by overcoming psychological obstacles to a healthier living [35].

### B. Pharmacological Interventions:

Pharmacological therapies are frequently utilized when lifestyle adjustments alone are insufficient. These treatments can focus on pathways involved in disease mechanisms. For instance, in addressing type 2 diabetes, drugs, such as metformin and insulin, are commonly prescribed to manage blood glucose levels. Likewise, antihypertensive medications play a crucial role in controlling high blood pressure, thereby lowering the risk of cardiovascular incidents [29]. The selection of pharmacological options depends on the patient's overall health status, severity of the condition, and potential side effects. It is vital to customize these treatments to meet individual requirements to enhance their effectiveness and reduce adverse effects [27].

### C. Surgical Interventions:

Surgical procedures are generally considered when other treatment options are ineffective or immediate action is required. For example, bariatric surgery is a recognized intervention for severe obesity and has demonstrated significant weight loss and improvement in obesity-related health issues. This procedure is particularly advantageous for individuals who have not achieved their desired results through lifestyle changes and pharmacological therapies. However, surgical alternatives carry inherent risks and require careful patient selection and postoperative care to ensure long-term success [72].

### D. Integration and Considerations:

Combining various strategies, as discussed above, often leads to the most favorable outcomes. For instance, merging lifestyle changes with pharmacological therapies can improve the management of chronic ailments, such as diabetes and hypertension. Additionally, presurgical lifestyle alterations can enhance surgical results and lower complications [27]. When formulating a treatment strategy, it is essential to consider patient preferences, possible side effects, and overall effect on quality of life.

## Emerging Research and Future Directions

Emerging investigations into innovative therapeutic targets, personalized medical strategies, and preventive measures are rapidly advancing, presenting promising avenues for future improvements in health care.

### A. Innovative Therapeutic Targets:

Recent investigations have uncovered numerous innovative therapeutic targets with the potential to transform treatment approaches. For example, some studies have underscored the significance of targeting specific molecular pathways that play a role in disease progression. One study highlighted the importance of certain proteins in cardiovascular illnesses, indicating that altering these proteins could create new therapeutic pathways [73,74]. Likewise, another study examined the discovery of distinct biomarkers in neurological conditions, which could act as targets for groundbreaking drug development [75,76]. These discoveries emphasize the necessity of comprehending disease mechanisms at the molecular level to create targeted therapies that are not only more effective, but also present fewer side effects.

### B. Personalized Medical Strategies:

Personalized medicine is increasingly being recognized as a revolutionary approach in healthcare, concentrating on customizing treatments based on individual patient attributes. Progress in genomics and bioinformatics has enabled the development of personalized treatment strategies. For instance, research has illustrated the effectiveness of genetic profiling in forecasting patient reactions to treatment in cardiovascular diseases and metabolic syndrome, thus enhancing treatment effectiveness while reducing adverse effects [77,78]. Furthermore, personalized medical strategies are being investigated in the management of chronic diseases, where patient-specific information is utilized to tailor treatment plans, leading to improved outcomes and higher patient satisfaction. These methodologies highlight the transition towards more individualized care, which is anticipated to bolster the accuracy and efficacy of medical interventions.

### C. Preventive Measures:

Preventive measures are vital for diminishing the occurrence and impact of diseases. Recent studies have focused on lifestyle changes and early interventions as essential elements of effective prevention strategies. Research has indicated that actions such as dietary modifications, increased physical activity, and smoking cessation can substantially lower the risk of developing chronic illnesses, such as diabetes and cardiovascular diseases [79]. Additionally, public health efforts aimed at raising awareness and educating communities about preventive practices have been effective in fostering healthier behaviors among populations [80-82]. These strategies underscore the significance of proactive health management and the role of preventive care in enhancing public health outcomes

## Conclusion

Metabolic Syndrome (MetS) is a cluster of metabolic abnormalities that significantly increases the risk of chronic conditions, such as type 2 diabetes, cardiovascular diseases, and other related disorders. It affects approximately 25% of the global population and is characterized by central obesity, high blood pressure, insulin resistance, high blood sugar, increased triglycerides, and low high-density lipoprotein (HDL) cholesterol levels. Studying and understanding MetS is crucial because of its significant public health implications and connection with severe health outcomes. MetS is associated with various health issues such as diabetes, neurodegenerative disorders, and non-alcoholic fatty liver disease. The pathophysiology of MetS involves the interplay of genetic and environmental factors, and understanding these mechanisms is vital for developing effective prevention and treatment strategies. Public health efforts and personalized preventive measures are essential for addressing the increasing prevalence of MetS. A current understanding of MetS is important for healthcare professionals to provide accurate diagnosis, effective management, and prevention of complications associated with MetS.

## Figures and Tables

**Table 1:** Criteria for metabolic syndrome defined by individual organization.

Organization	Criteria
**World Health Organization (WHO) Criteria**	Central obesity (waist circumference > 94 cm for men and > 80 cm for women) plus any two of the following: Raised triglycerides (≥ 150 mg/dL) ; Reduced HDL cholesterol (< 40 mg/dL for men and < 50 mg/dL for women) ; High blood pressure (≥ 130/85 mmHg) ; Increased fasting glucose (≥ 110 mg/dL)
**National Cholesterol Education Program (NCEP) / Adult Treatment Panel III (ATP III)**	Diagnosis requires at least three of the following five criteria: Abdominal obesity (waist circumference > 102 cm for men and > 88 cm for women); Increased triglycerides (≥ 150 mg/dL) ; Reduced HDL cholesterol (< 40 mg/dL for men and < 50 mg/dL for women) ; High blood pressure (≥ 130/85 mmHg) ; Increased fasting glucose (≥ 100 mg/dL)
**International Diabetes Federation (IDF)**	Central obesity (waist circumference ≥ 94 cm for men and ≥ 80 cm for women) plus any two of the following: Increased triglycerides (≥ 150 mg/dL) ; Reduced HDL cholesterol (< 40 mg/dL for men and < 50 mg/dL for women) ; High blood pressure (≥ 130/85 mmHg) ; Increased fasting glucose (≥ 100 mg/dL)
